# The Future Diabetes Mortality: Challenges in Meeting the 2030 Sustainable Development Goal of Reducing Premature Mortality from Diabetes

**DOI:** 10.3390/jcm14103364

**Published:** 2025-05-12

**Authors:** Kaustubh Wagh, Alexander Kirpich, Gerardo Chowell

**Affiliations:** 1 Department of Population Health Sciences, School of Public Health, Georgia State University, Atlanta, GA 30303, USA; 2 Department of Applied Mathematics, Kyung Hee University, Yongin 17104, Republic of Korea

**Keywords:** diabetes, forecast, global

## Abstract

**Objective:** This study seeks to forecast the global burden of diabetes-related mortality by type, age group, WHO region, and income classification through 2030, and to assess progress toward Sustainable Development Goal (SDG) 3.4, which aims to reduce premature mortality (among people age 30–70 years) from noncommunicable diseases (including diabetes) by one-third. **Methods:** We analyzed diabetes mortality data from the Institute for Health Metrics and Evaluation, Global Burden of Disease 2019, covering 30 years (1990–2019). Using this historical dataset, we generated 11-year prospective forecasts (2020–2030) globally and stratified by diabetes type (type 1, type 2), age groups, WHO regions, and World Bank income classifications. We employed multiple time series and epidemic modeling approaches to enhance predictive accuracy, including ARIMA, GAM, GLM, Facebook’s Prophet, n-sub-epidemic, and spatial wave models. We compared model outputs to identify consistent patterns and trends. **Results:** Our forecasts indicate a substantial increase in global diabetes-related mortality, with type 2 diabetes driving the majority of deaths. By 2030, annual diabetes mortality is projected to reach 1.63 million deaths (95% PI: 1.48–1.91 million), reflecting a 10% increase compared to 2019. Particularly concerning is the projected rise in mortality among adults aged 15–49 and 50–69 years, especially in Southeast Asia and low- and middle-income countries. Mortality in upper-middle-income countries is also expected to increase significantly, exceeding a 50% rise compared to 2019. **Conclusions:** Diabetes-related deaths are rising globally, particularly in younger and middle-aged adults in resource-limited settings. These trends jeopardize the achievement of SDG 3.4. Urgent action is needed to strengthen prevention, early detection, and management strategies, especially in Southeast Asia and low-income regions. Our findings provide data-driven insights to inform global policy and target public health interventions.

## 1. Introduction

Diabetes mellitus, a chronic metabolic disorder characterized by high blood sugar levels, is a growing global health crisis. The International Diabetes Federation estimates that 537 million adults worldwide currently live with diabetes, a number projected to rise to 783 million by 2045 [1]. Recent global burden assessments show a significant increase in diabetes prevalence during the past three decades, with the steepest rises occurring in low- and middle-income countries [2,3,4]. Diabetes occurs when the pancreas produces insufficient insulin or the body cannot effectively use it, leading to hyperglycemia. Prolonged high blood glucose levels increase the risk of severe complications such as heart attacks, strokes, kidney failure, and limb amputations. Diabetes is classified into three types. Type 1 diabetes results from autoimmune destruction of insulin-producing beta cells, with no known cause or prevention; type 2 diabetes, which accounts for more than 95% of all cases, occurs when the body develops insulin resistance, often due to obesity, physical inactivity, and genetic predisposition [5]. Unlike type 1, type 2 diabetes is largely preventable. Gestational diabetes develops during pregnancy, increasing the risk of complications for both mother and child and predisposing both to type 2 diabetes later in life.

In recent years, diabetes-related deaths have surged globally, making it one of the leading causes of mortality [6]. According to the World Health Organization (WHO), diabetes was the eighth leading cause of death in 2019, responsible for 1.6 million deaths [7,8]. Mortality trends indicate a 70% increase in diabetes-related deaths since 1990, with the greatest burden observed in Southeast Asia and Sub-Saharan Africa. The geographic and demographic distribution of diabetes varies significantly [9]. Developing countries, particularly in Asia and Africa, are experiencing a diabetes epidemic fueled by rapid urbanization, sedentary lifestyles, and changing diets [10]. Socioeconomic disparities exacerbate the crisis, as limited healthcare access and financial constraints lead to poor disease management, particularly in lower-income populations. Diabetes is more common in men than in women, and prevalence increases with age. However, a concerning rise in younger adult cases (ages 15–49) suggests future health and economic implications.

Given the rising burden of diabetes mortality, robust forecasting is crucial for informing public health policies, optimizing resource allocation, and developing targeted intervention strategies. Previous forecasting studies have used traditional statistical models such as autoregressive integrated moving average (ARIMA) and machine learning-based frameworks [11,12]. However, many of these models fail to account for socioeconomic heterogeneity, spatial dependencies, and the influence of multiple epidemic waves. Accurate forecasting of diabetes mortality is essential to guide policymakers in implementing effective prevention programs. It can also help global health initiatives understand progress toward the WHO’s Sustainable Development Goal (SDG) 3.4, which aims to reduce premature mortality from noncommunicable diseases, including diabetes, by one-third by 2030 [13,14,15].

Among WHO regions, Southeast Asia region has the highest diabetes mortality rates, particularly in India, Pakistan, and Bangladesh, where rapid urbanization and limited healthcare infrastructure contribute to rising deaths. Forecasting mortality in this region is crucial for understanding the global trajectory of diabetes mortality by 2030.

Similarly, the diabetes burden is staggering in both low- and middle-income countries (LMICs) and upper-middle income countries (UMICs). Urbanization, dietary shifts, and inadequate healthcare access are key drivers of the crisis in LMICs, leading to severe economic consequences, including lost productivity and rising healthcare costs. Even in UMICs, where healthcare infrastructure is relatively better, managing diabetes remains complex and costly due to its chronic nature.

This study aims to address the global burden of diabetes mortality by employing a novel forecasting approach that integrates sub-epidemic and spatial wave models. Our model incorporates diabetes type, age groups, WHO regions, and World Bank income classifications. To our knowledge, this is the first study to use Global Burden of Disease 2019 data for comprehensive global diabetes mortality forecasting. Historically, diabetes mortality has been highest among adults older than 50 years of age. However, with increasing cases among younger age groups (15–49 years), there is an urgent need to evaluate future mortality patterns across age demographics. We focus on forecasting mortality under 70 years of age, given its importance in measuring premature noncommunicable disease deaths per SDG 3.4.

## 2. Materials and Methods

### 2.1. Data Collection and Preparation

We accessed publicly available diabetes mortality data from Our World in Data through https://ourworldindata.org/data and downloaded it on 23 March 2024. The original source of data is the Institute for Health Metrics and Evaluation, Global Burden of Disease 2019, which was retrieved on 22 September 2021, according to Our World in Data [16]. The mortality (number of deaths caused by diabetes) data were available for the years 1990 to 2019 (30 years) with annual frequency and segregated by type, age groups, WHO regions, and WB’s income classification. We used all 30 years of data, so we set the calibration period to 30 years and the forecasting horizon between 2020 and 2030 (11 years). Data were preprocessed using Microsoft Excel pivot tables. All forecasting analyses were conducted using Rstudio, Posit Software, Boston, MA, USA and MATLAB, MathWorks Inc., Natick, MA, USA. Specifically, we used the StatModPredict dashboard (developed in RStudio v. 4.3.2) to generate forecasts from the ARIMA, GAM, GLM, and Prophet models (Taylor & Letham, 2018). For the ensemble n-sub-epidemic modeling framework, we employed the SubEpiPredict toolbox (subEpiPredict toolbox) implemented in MATLAB (R2023b) [17]. These modeling toolboxes were developed to facilitate reproducible, user-friendly forecasting workflows. A brief description of the models is provided below and a comprehensive overview of each model is included in the Supplementary Materials.

### 2.2. Forecasting Models

#### 2.2.1. ARIMA (Autoregressive Integrated Moving Average)

ARIMA is a time series model that builds forecasts by identifying patterns in past mortality data [18]. It combines three components: trends over time (autoregression), smoothing of random fluctuations (moving average), and differencing to stabilize the data. ARIMA works best for short-term forecasting when historical trends are stable and linear [19]. However, it is less effective when mortality patterns shift suddenly due to interventions or emerging risk factors [19].

#### 2.2.2. GAM (Generalized Additive Model)

GAMs extend traditional regression models that allow for flexible, nonlinear relationships between time and mortality outcomes [20]. Instead of fitting a straight line, GAMs use smooth curves to model long-term trends in diabetes deaths [21]. This makes them well suited for capturing gradual changes in mortality that may result from evolving lifestyle behaviors, treatment access, or demographic shifts.

#### 2.2.3. GLM (Generalized Linear Model)

GLMs are classic statistical models used to relate a response variable (like mortality count) to one or more predictors (such as time). They assume a specific relationship—often linear—between the predictors and the outcome, and use a distribution suited for the type of data (e.g., Poisson or normal) [22]. GLMs are straightforward, interpretable, and effective when trends are relatively smooth and consistent.

#### 2.2.4. Prophet (Developed by Meta/Facebook)

Prophet is a decomposable time series model that breaks down mortality trends into three components: an overall trend, seasonal effects (e.g., annual patterns), and abrupt changes due to major events or interventions [23]. It is highly automated and designed to handle missing data and outliers well [24]. Prophet is especially useful for longer-term forecasting where trends may shift, making it helpful for identifying inflection points in chronic disease burden.

#### 2.2.5. n-Sub-Epidemic Model

This model interprets mortality trends as a series of overlapping “sub-epidemics”, each representing a surge or phase in diabetes deaths [25,26]. Each sub-epidemic has its own timing and intensity, allowing the overall model to capture complex mortality dynamics—such as plateaus or multiple peaks—often seen in real-world chronic disease progression. It is particularly powerful in reflecting regional differences or the cumulative effects of repeated disruptions.

#### 2.2.6. Ensemble Sub-Epidemic Models (Unweighted and Weighted)

These models combine forecasts from the top-performing sub-epidemic models. The unweighted ensemble gives equal importance to each model, while the weighted version gives more influence to models with better historical fit [27]. This ensemble approach improves robustness by balancing strengths across models and helps produce more reliable forecasts with quantified uncertainty.

**Figure 1:**
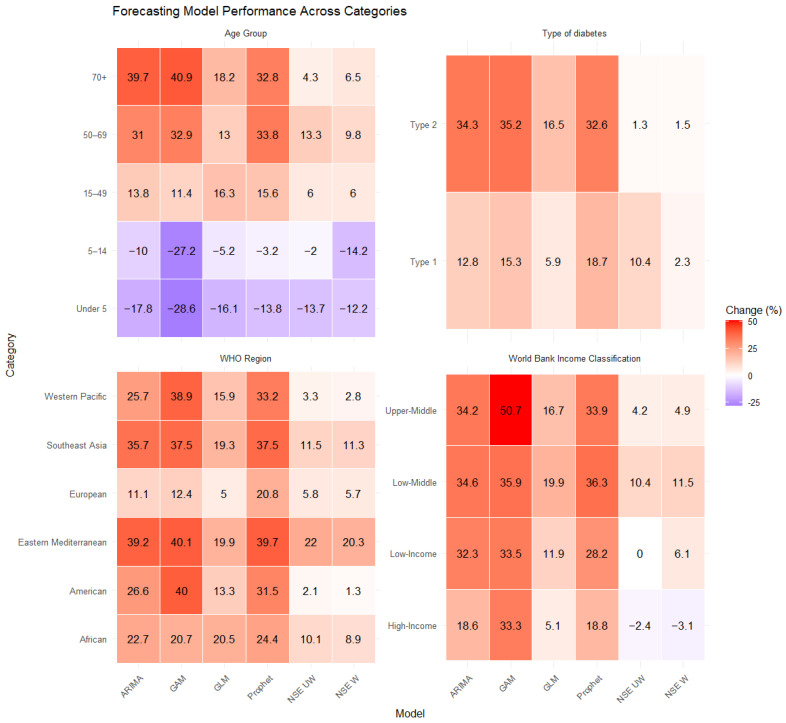
Percentage changes in the number of deaths from diabetes from 2019 to 2030 globally by age group, type of diabetes, WHO regions, and World Bank Income classification. The panel shows the relative percentage change from the last year of data used during the model calibration process (2019) to the forecasted median number of deaths from diabetes in 2030. The panel shows results from six different models—ARIMA, GAM, GLM, Prophet, n-sub-epidemic ensemble unweighted (NSE UW), and n-sub-epidemic ensemble weighted (NSE W). Purple tiles indicate forecasted decreases in mortality between 2019 and 2030, and red tiles indicate forecasted increases in mortality during same period. Darker colors indicate a greater percentage change in either direction (i.e., negative or positive). The values within each tile correspond to the relative percentage change in number of deaths from diabetes from 2019 to 2030.

**Figure 2:**
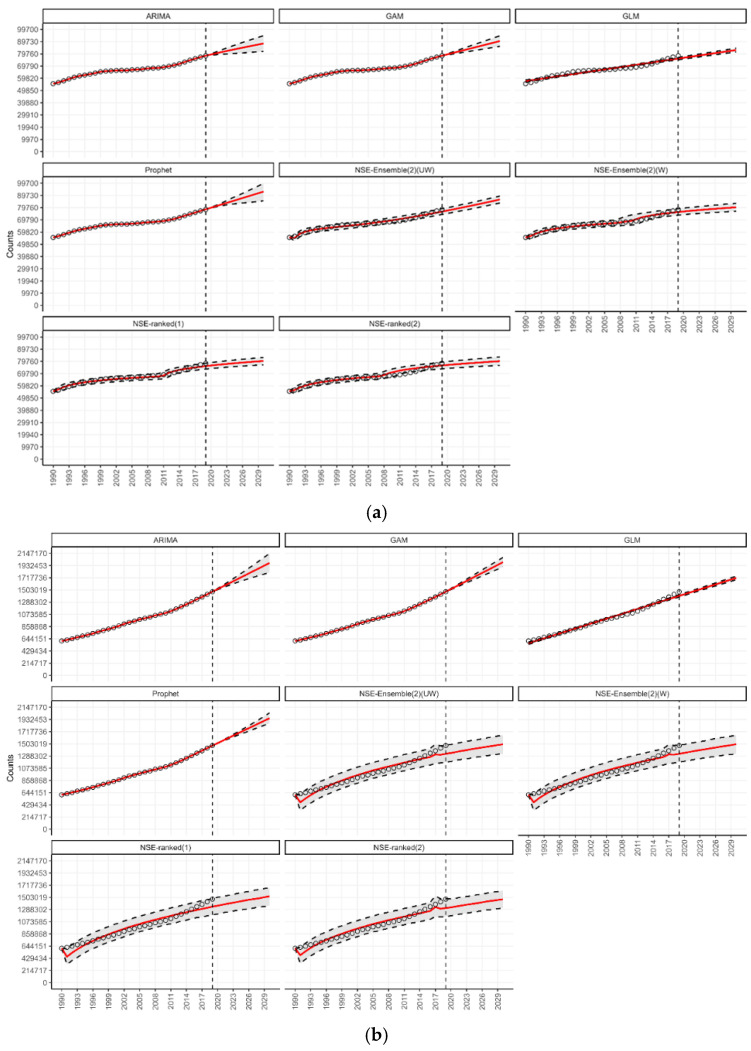
(**a**) Forecasts for the number of deaths from type 1 diabetes globally from 2020 to 2030. (**b**) Forecasts for the number of deaths from type 2 diabetes globally from 2020 to 2030.

**Figure 3:**
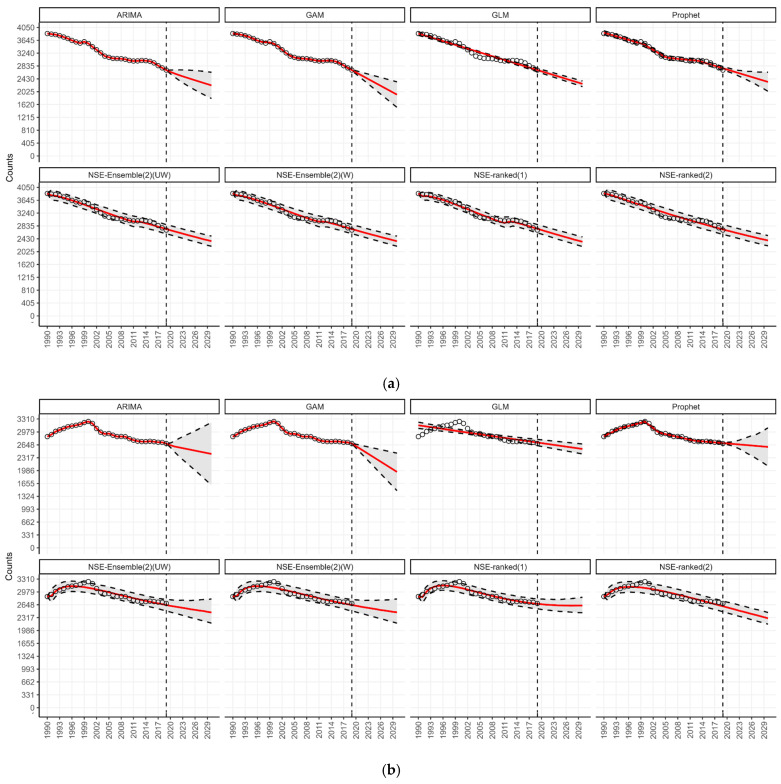

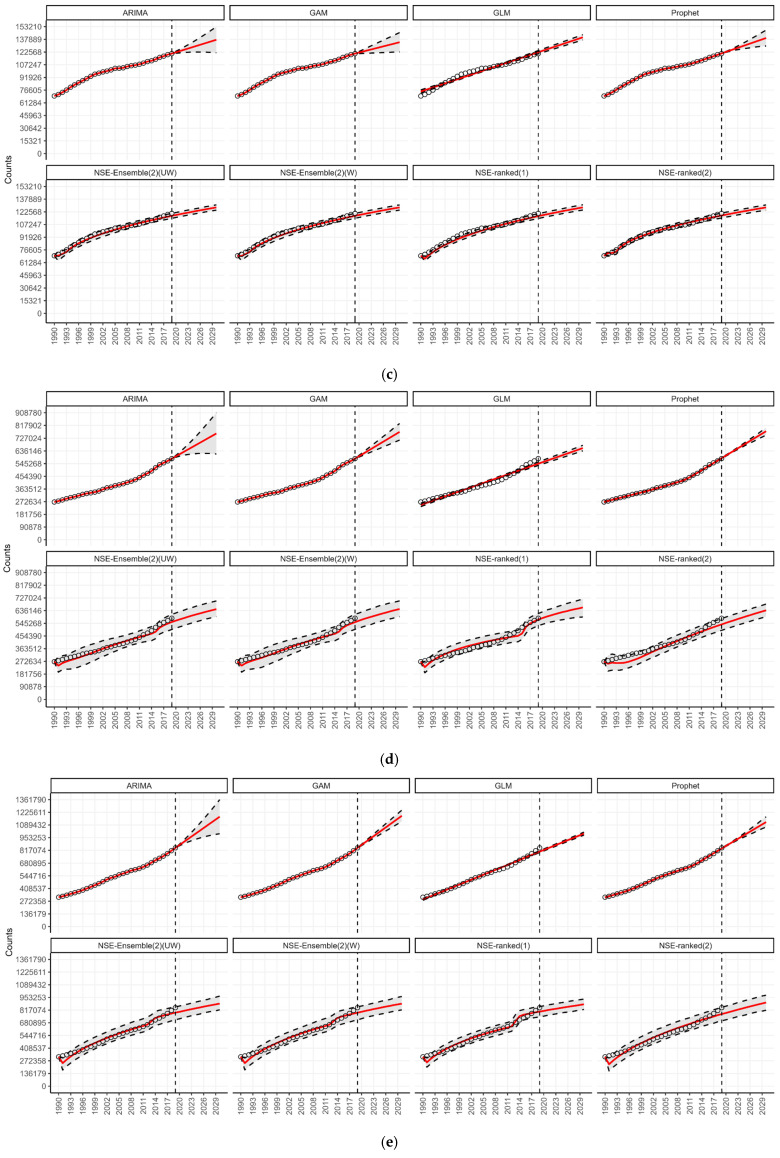
(**a**) Forecasts for the number of deaths from diabetes among age group under 5 years globally from 2020 to 2030. (**b**) Forecasts for the number of deaths from diabetes among age group 5–14 years globally from 2020 to 2030. (**c**) Forecasts for the number of deaths from diabetes among age group 15–49 years globally from 2020 to 2030. (**d**) Forecasts for the number of deaths from diabetes among age group 50–69 years globally from 2020 to 2030. (**e**) Forecasts for the number of deaths from diabetes among age group 70+ years globally from 2020 to 2030.

**Figure 4:**
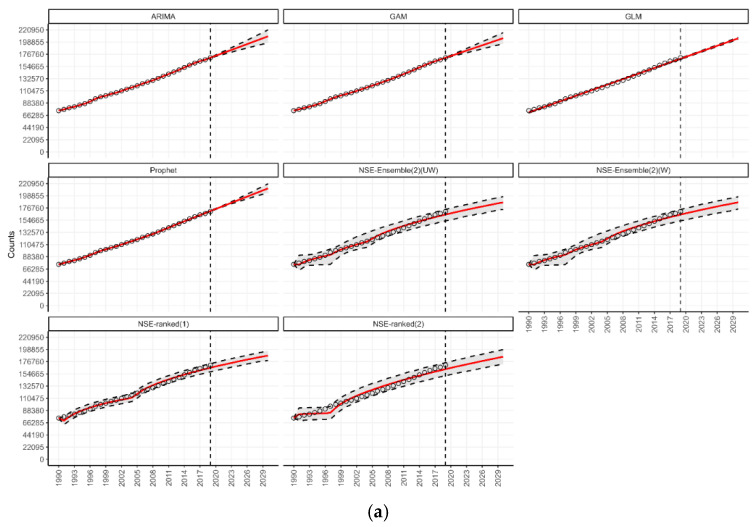

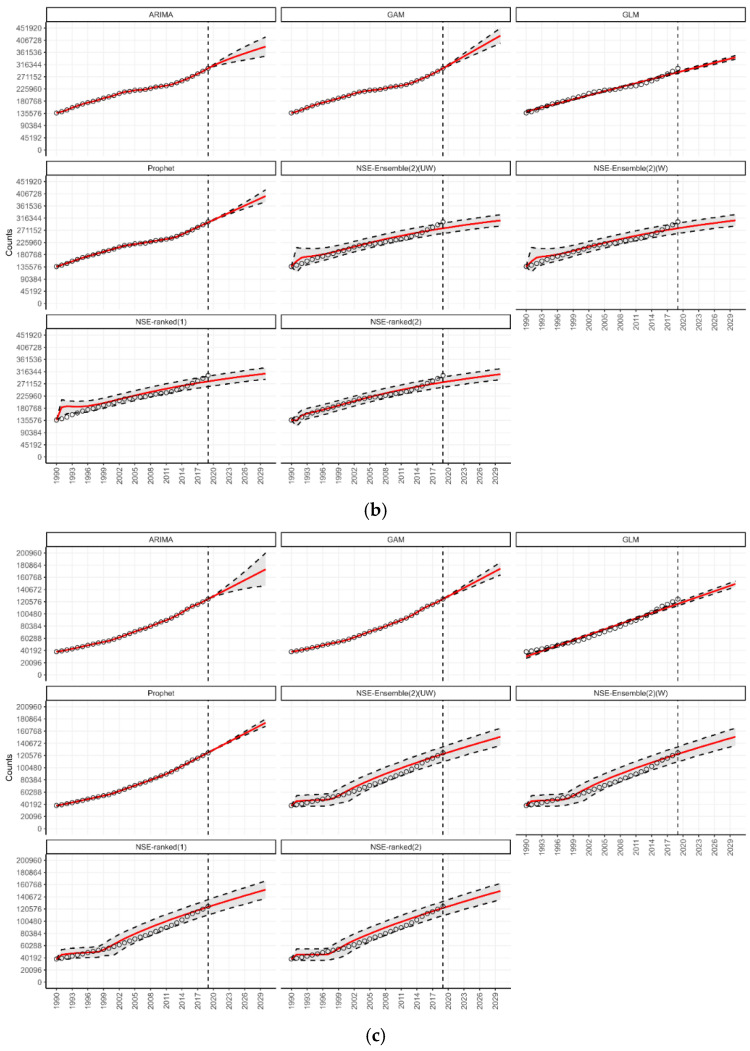

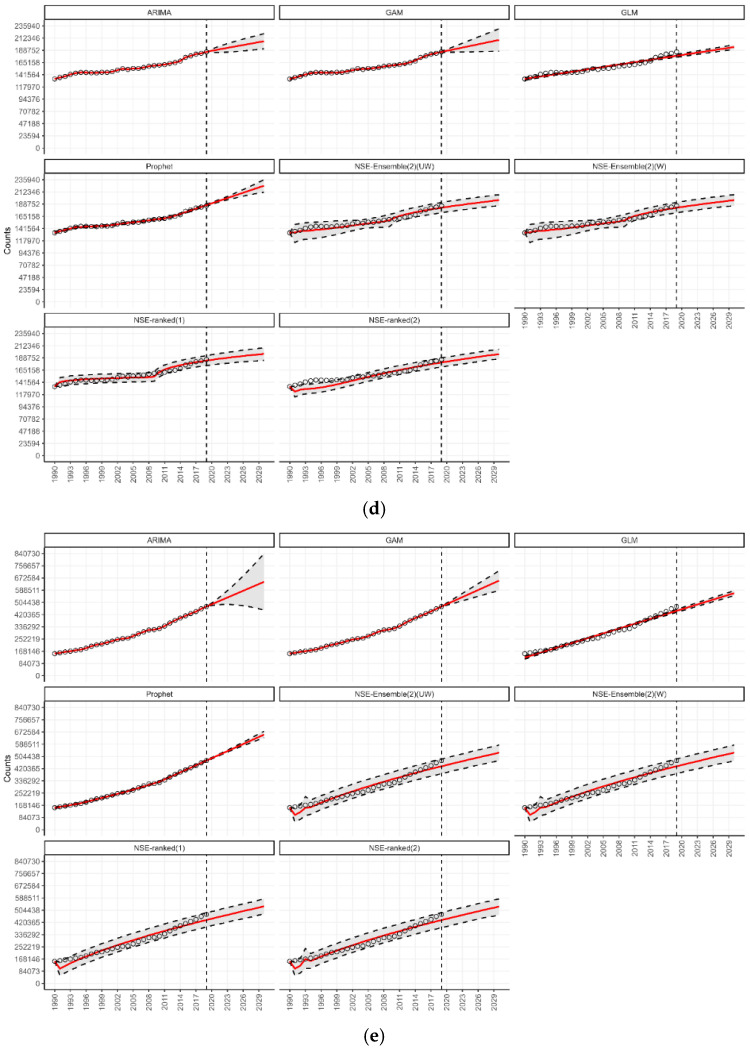

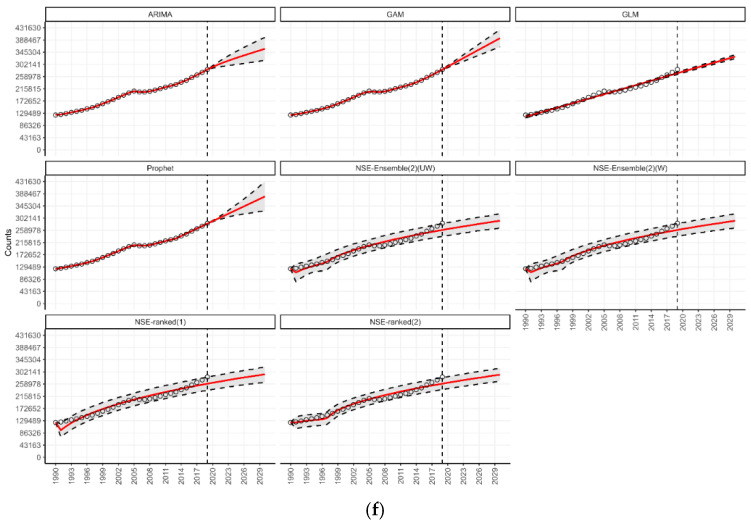
(**a**) Forecasts for the number of deaths from diabetes in the African region from 2020 to 2030. (**b**) Forecasts for the number of deaths from diabetes in the American region from 2020 to 2030. (**c**) Forecasts for the number of deaths from diabetes in the Eastern Mediterranean region from 2020 to 2030. (**d**) Forecasts for the number of deaths from diabetes in the European region from 2020 to 2030. (**e**) Forecasts for the number of deaths from diabetes in the Southeast Asia region from 2020 to 2030. (**f**) Forecasts for the number of deaths from diabetes in the Western Pacific region from 2020 to 2030.

**Figure 5:**
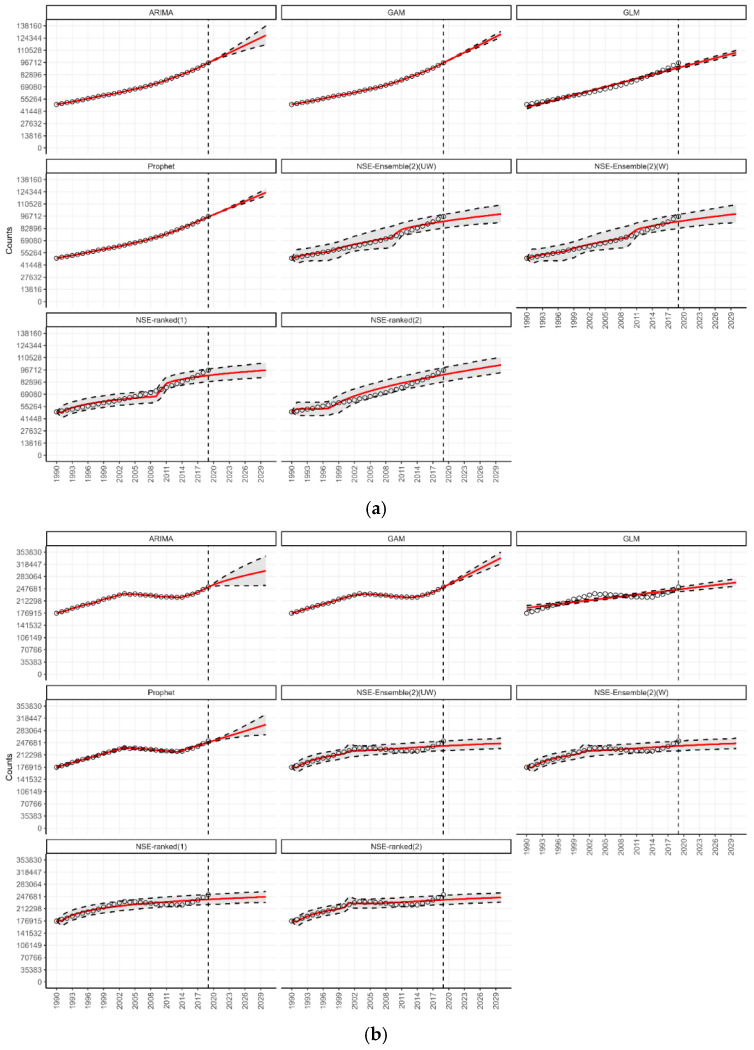

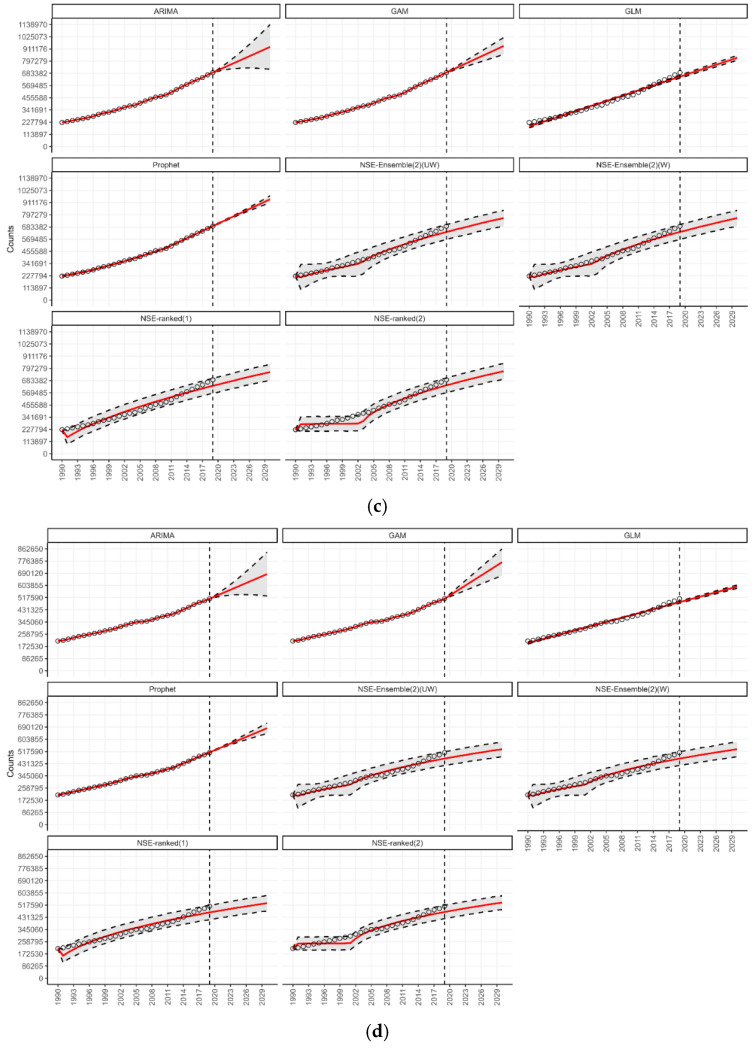
(**a**) Forecasts for the number of deaths from diabetes in low-income countries from 2020 to 2030. (**b**) Forecasts for the number of deaths from diabetes in high-income countries from 2020 to 2030. (**c**) Forecasts for the number of deaths from diabetes in low-middle-income countries from 2020 to 2030. (**d**) Forecasts for the number of deaths from diabetes in upper-middle-income countries from 2020 to 2030.

Under the n-sub-epidemic modeling framework, we developed several models, including the ensemble unweighted (NSE UW), the ensemble weighted (NSE W), and the top two individually ranked models (NSE ranked (1) and NSE ranked (2)). We focused on NSE UW and NSE W through Table 1 and Figure 1, excluding individual NSE-ranked models, as they are encapsulated within the ensemble approaches. The ensemble models (NSE UW, NSE W) are superior to individual models (NSE ranked (1), NSE ranked (2)) as they improve forecasting performance by systematically incorporating the predictive accuracy of individual models [27]. However, for transparency and to show the individual models that informed our ensemble approaches, we presented the results of NSE-ranked (1) and NSE-ranked (2) models in panel Figure 2, Figure 3, Figure 4 and Figure 5. The panel figures (Figure 2, Figure 3, Figure 4 and Figure 5) illustrate the forecast derived from numerous models. The black circles to the left of the vertical dashed line denote observed diabetes deaths up to the year 2019, which marked the end of the calibration period. The red solid line marks the trajectory of the best-fit model, while black dashed lines represent the 95% prediction intervals. The vertical dashed black line signifies the start of the forecast period (2020–2030).

### 2.3. Model Evaluation

Models were evaluated using a set of performance metrics that measure point and probabilistic forecast accuracy simultaneously [27]. Models were evaluated based on mean square error and mean absolute error, which quantify the deviation between predicted and the observed values for a given point in time. In this context, smaller values indicate better model performance. The reliability of uncertainty was evaluated based on the proportion of observations fall in the predicted intervals, with 95% P.I coverage where higher values ideally estimate better calibrated uncertainty [28]. In addition, WIS provides balance between sharp and out-of-range predictions due to penalizing prediction intervals that are too wide and observations that fall outside predicted range, thus sustaining an overall balance between forecast sharpness and calibration [25].

### 2.4. Quantifying Forecast

We quantified the trends in each model. We used the percentage change formula to calculate the relative change (%) in the number of diabetes-related deaths between 2019 and 2030. The formula was (Forecast (2030) − Observed (2019)/Observed (2019)) × 100. Here, forecast (2030) refers to the forecasted number of diabetes-related deaths in 2030, and observed (2019) refers to the observed number of diabetes-related deaths in 2019, which is the last year of available data. We calculated the overall diabetes-related mortality in 2030 by first calculating the mean forecast across all models for each diabetes type separately and then aggregating these type-specific estimates to obtain the total burden.

## 3. Results

### 3.1. Forecasting by Type of Diabetes

Our 11-year-ahead forecasts from the six different models indicate an increase in the number of deaths from type 1 diabetes (Figure 1). Although the magnitude of deaths from type 1 diabetes is significantly lower than that of type 2 diabetes, there was a rise in type 1-related mortality between 1990 and 2019 (Figure 2a,b). In the Prophet and GAM models, a total of 92.9 thousand and 90.3 thousand (95% PI 85.6, 100.3, and 95% PI 86.0, 94.6) type 1 diabetes-related deaths could be added globally per year by 2030, respectively (Table 1). This would be more than a 15 percent rise in the annual type 1 diabetes-related deaths compared to 2019. Overall, the number of deaths from diabetes doubled between 1990 and 2019. Most diabetes-related deaths are attributed to type 2 diabetes and people greater than 50 years of age (Figure 1). Based on the global trend, our 11-year-ahead forecasts from the different models continue to support a significant increase in the number of deaths from type 2 diabetes (Figure 1). According to our forecasts from the GAM, ARIMA, and Prophet models, 1.9 million or more (95% PI: (1.9; 2.1), (1.8; 2.1), (1.8; 2.0), respectively) deaths could be added globally per year by 2030 (Table 1). This would be more than a 30 percent rise in the annual type 2 diabetes-related deaths compared to 2019.

### 3.2. Forecasting by Age Groups

Our 11-year-ahead forecasts from the eight different models continue to support an increase in the number of deaths from diabetes for age groups 15–49 and 50–69 years (Figure 3c,d). According to our forecasts from the Prophet and GLM models, approximately 140 thousand (95% PI: 137.3; 143.6, and 95% PI: 130.0; 149.1) deaths could be added globally per year by 2030 in the age group 15–49 (Table 1). This would be more than a 15 percent rise in the annual diabetes-related deaths in this age group compared to 2019 (Figure 1). Similarly, forecasts from the Prophet and GAM models suggest a total of 778 thousand (95% PI: 750.5; 801.5, and 95% PI: 714.5; 831.7) deaths could be added globally per year by 2030 in the age group 50–69 (Table 1). This would be more than a 30 percent rise in the annual diabetes-related deaths in this age group compared to 2019 (Figure 1). The forecasting details of all age groups are included in Figure 3a–e.

### 3.3. Forecasting by WHO Regions

Our forecast suggests the number of deaths from diabetes will be highest in the Southeast Asia region, followed by the Americas and Western Pacific regions (Table 1). The percentage of change in the number of deaths in 2030 compared to 2019 would be the highest in the Eastern Mediterranean region, which is consistent across various models (Figure 1). According to our forecasts from the Prophet and GAM models, a total of 655 thousand (95% PI 630.2; 677.8, and 95% PI 586.7; 722.1) deaths could be added globally per year by 2030 in the Southeast Asia region (Table 1). This would be greater than a 35 percent rise in the annual diabetes-related deaths in this region compared to 2019. Similarly, forecasts in the Americas from the GAM model suggest that approx. 424 thousand (95% PI 395.9; 451.9) deaths could be added globally per year by 2030 (Table 1). This would be more than a 40 percent rise in the annual diabetes-related deaths in this age group compared to 2019. The forecasting details for all WHO regions are shown in Figure 4a–f.

### 3.4. Forecasting for Countries by World Bank Income Classification

The forecast indicates countries in the World Bank’s low-middle income classification expected to report the highest number of deaths from diabetes (approx. between 763 and 942 thousand, depending upon the model) by 2030, followed by upper-middle and high-income countries (Table 1). According to our forecasts from the Prophet and GAM models, a total of 942 thousand (95% PI 909.6; 973.8, and 95% PI 860.0; 1017.5) deaths could be added globally per year by 2030 in low-middle income countries (Table 1). This would be greater than a 35 percent rise in the annual diabetes-related deaths in these countries compared to 2019. Similarly, forecasts in upper-middle income countries from the GAM model suggest that a total of 768 thousand (95% PI 673.7; 862.7) deaths could be added globally per year by 2030 (Table 1). This would be more than a 50 percent rise in the annual diabetes-related deaths in these countries compared to 2019. The forecasting details of countries in all income groups are shown in Figure 5a–d.

### 3.5. Forecasting in the Context of SDG 3.4

The Sustainable Development Goal (SDG) 3.4 intends to reduce the premature mortality (between the ages of 30 and 70) from four noncommunicable diseases, including cancers, cardiovascular diseases, chronic respiratory diseases, and diabetes, by one-third by 2030 compared to 2015 levels. To assess progress toward SDG target 3.4, we compared projected diabetes-related mortality in 2030 with baseline 2015 levels, specifically focusing on premature deaths in working-age populations (15–49 and 50–69 years) rather than the standard age range of 30–70 years for SDG 3.4, due to limitations in the available age group data. By 2030, diabetes-related mortality is projected to rise 23% for ages 15–49 and 51% for ages 50–69 compared to 2015 levels. Between 2015 and 2030, the proportion of all diabetes-related deaths occurring in younger adults (15–49 years) is expected to rise slightly, from 8.3% to 8.6%, while the proportion in middle-aged adults (50–69 years) is projected to increase substantially, from 37.8% to 47.7%. These trends directly contradict SDG 3.4, which targets a one-third reduction in premature mortality from noncommunicable diseases by 2030.

### 3.6. Summary of Main Findings

Global diabetes-related mortality is projected to rise significantly by 2030, undermining progress toward the Sustainable Development Goal (SDG) 3.4, which aims to reduce premature mortality from noncommunicable diseases by one-third. Type 2 diabetes is the primary driver of the projected mortality burden, although increases in type 1 diabetes-related deaths are also forecasted. Forecasting models consistently predict an upward trend, especially in low- and middle-income countries and among younger adult populations. Specifically, global annual diabetes deaths are forecasted to increase by more than 10% by 2030, reaching approximately 1.63 million deaths (95% Prediction Interval: 1.48–1.91 million). Type 2 diabetes mortality is expected to rise by greater than 30% globally compared to 2019, with some models forecasting an addition of nearly 2 million deaths per year by 2030. Meanwhile, type 1 diabetes mortality may increase by more than 15%, with forecasts indicating an additional 90,000 to 93,000 deaths annually by 2030. The ensemble n-sub-epidemic models (NSE-UW and NSE-W) better captured complex epidemic trajectories by combining multiple model signals. Adults aged 15–49 and 50–69 are projected to experience the most significant increases in diabetes-related mortality, which raises serious concerns for public health systems targeting premature mortality. From an economic and geographic perspective, lower-middle-income countries (LMICs) face the steepest increases, with diabetes mortality projected to rise between 10.4% and 36.3% by 2030. Southeast Asia and the Eastern Mediterranean region show the highest projected increases in diabetes mortality, followed by Sub-Saharan Africa and Oceania. In contrast, high-income countries show smaller increases (~11.6%) than low-income countries (~18.6%), which likely reflect differences in healthcare access, diagnosis, and disease management.

## 4. Discussion

Researchers have utilized various forecasting models, including ARIMA, GAM, and machine learning-based approaches, to predict trends in diabetes-related mortality [8,12]. However, to our knowledge, this study is the first to integrate multiple forecasting models, including the n-sub-epidemic (NSE) model, providing a comprehensive comparative analysis of different methodologies. This multimodel approach enhances our understanding of diabetes mortality trajectories by capturing diverse trend patterns and model uncertainties.

Among the models tested, ARIMA produced the widest 95% prediction intervals (PI), suggesting higher forecast variability. At the same time, GLM generated the narrowest PIs, indicating more stable but potentially less flexible predictions. The NSE models provided balanced performance, effectively capturing both short-term fluctuations and long-term mortality trends.

Our projections indicate that by 2030, annual global deaths from diabetes will rise to 1.63 million (95% PI: 1.48–1.91 million), representing a 10% increase compared to 2019. This surge is predominantly driven by type 2 diabetes and mortality among individuals aged 50 and older. Particularly concerning is the projected 5% rise in diabetes-related deaths among young adults (15–49 years), which aligns with emerging evidence about high diabetes burden within this demographic [29,30,31]. Research has shown that younger patients with type 2 diabetes experience higher mortality risks compared to older patients, with one study reporting 100–200% increased mortality among patients under 55 years compared to only 30–40% excess risk in those aged 65–74 years [32]. Adolescents with type 2 diabetes have 1.5 times higher short-term mortality than those with type 1 [33]. CDC projections suggest that the number of type 2 diabetes cases among adolescents may rise as much as 700% by the year 2060, even if the incidence remains stable [34], which shows diabetes burden is shifting toward younger populations. This trend poses significant public health and economic challenges, reinforcing concerns that the SDG 3.4 target of reducing premature deaths from noncommunicable diseases including diabetes by one-third will not be met by 2030 [10].

The increasing global burden of diabetes is well documented, with incidence and prevalence rising steadily since 1990 [2,7,35]. Projections suggest that by 2030, the number of people living with diabetes could increase by 25–51% [7,35,36]. A previous ARIMA-based study forecasting diabetes mortality through 2025 estimated that diabetes-related deaths would reach 1.59 million, closely aligning with our findings [2]. These increases are primarily attributed to type 2 diabetes, with metabolic and behavioral risk factors, including obesity, poor diet, smoking, and sedentary lifestyles, as the major contributors.

Disparities in diabetes burden across WHO regions reflect complex interaction between socioeconomic factors, healthcare access, and epidemiological transitions [37]. The greatest concern lies within the Southeast Asia region (SEAR) and the Eastern Mediterranean region (EMR). They expect the highest projected diabetes mortality, followed by Oceania and Sub-Saharan Africa. This aligns with the 2050 projection that North Africa and the Middle East will surpass all other super-regions with diabetes prevalence at 16.8% [3]. The Western Pacific region (WPR) is suffering from an unprecedented increase in diabetes burden due to urban migration and lifestyle alterations [38]. The European region (EUR) does not face the same level of challenge. However, it still suffers from the rising burden of diabetes. The African region (AFR) continues to show a low prevalence of diabetes, but the healthcare systems limitations continue to pose a challenge [39]. The region of the Americas (AMR) displays high-income North America and Latine heterogeneities [40]. In the United States, diabetes prevalence is expected to rise by 54% by 2030, leading to a 38% increase in annual deaths and a 53% escalation in related healthcare costs [11]. Such disparities highlight the urgent need for tailored strategies to strengthen diabetes prevention and care while managing healthcare infrastructure, particularly in areas where the burden is rising rapidly but resources are severely constrained [41].

Similarly, the impact of diabetes differs significantly within various World Bank income regions. Our most worrying projection is for lower-middle-income countries (LMICs), which suggest a 10.4% to 36.3% increase in diabetes mortality by 2030 compared to 2019. This aligns with studies that indicate yearly increase in age-standardized death rates of 1.3% from type 2 diabetes in LMICs [42]. Overall socioeconomic development and diabetes mortality rates show an inverse U-shaped relationship, which indicates middle-income regions are disproportionately burdened [2]. Interestingly, when compared to low-income regions, high-income countries tend to have a higher prevalence of diabetes, but with significantly better disease management outcomes [43]. Our findings align with this, which shows an average percentage of change in mortality between 2019 and 2030 across all models in high-income countries that is lower than low-income countries (11.6% vs. 18.6%). This highlights the gap in undiagnosed cases, and capacities of countries, especially LICs or LMICs, for testing, diagnosing, and treating of diabetes [44,45]. These gaps represent a significant mortality burden with diabetes accounting for 3.4 million deaths in the year 2024 alone, which breaks down to about one death every nine seconds [46]. This suggests need for a global response to enhance prevention and management strategies [47].

### 4.1. Implications for Public Health and Healthcare Systems

These projections highlight an urgent need for strengthened global diabetes prevention and treatment initiatives. Health policies should focus on expanding diabetes screening programs in LMICs and target prevention programs at younger age groups (15–49), given rising mortality in this cohort. If current trends continue, diabetes-related mortality will not only rise but will place an increasing burden on healthcare systems, economies, and society. According to the International Diabetes Federation, the number of type 2 diabetes cases alone could reach 552 million by 2030. This surge will exacerbate existing challenges, including increased healthcare expenditures, reduced workforce productivity, and higher demands on medical infrastructure. The financial implications of this epidemic are staggering. In the United States, total annual medical and societal costs related to diabetes are projected to exceed 622 billion USD by 2030 [11]. Beyond direct healthcare costs, diabetes is a leading risk factor for cardiovascular diseases, further increasing mortality and healthcare expenditures.

### 4.2. Limitations

This forecasting analysis has limitations. First, the models used rely on the availability and precision of historical data, which may not accurately represent future scenarios. Any significant shifts in major health policies may impact these forecasts. Second, every forecasting model has some limitations. ARIMA assumes linear relationships and stationarity, which work well for forecasting diabetes mortality in the short term, but they could struggle to capture nonlinear progression patterns within the disease and structural shifts resulting from novel treatments or policy changes. GAMs can model nonlinear relationships, but they can fail to capture complex temporal dependencies in mortality trends, especially with gaps in longitudinal data. While adept with capturing seasonal patterns of diabetes deaths, Prophet’s decomposition approach is poor at handling changes in mortality when driven by new interventions. Though n-sub-epidemic models are theoretically appealing for capturing distinct epidemic waves, they may overfit minor fluctuations in the data, particularly when noise levels are high. However, model selection based on the corrected Akaike information criterion (AICc) helps mitigate this risk by favoring models with lower complexity (e.g., fewer sub-epidemics) rather than higher complexity (e.g., more sub-epidemics). Third, the reliance on historical data up to 2019 does not include the healthcare disruptions caused by the COVID-19 pandemic, including but not limited to delayed diagnoses and interrupted diabetes care. However, these disruptions may have increased diabetes-related mortality [48]. Fourth, there is a possibility of inaccuracies in GBD’s estimates of diabetes mortality, especially in lower-middle-income countries, due to incomplete vital registration systems with significant unrecorded deaths, systematic misclassification of diabetes as a cause of death, underdiagnosed cases due to insufficient diagnostic capacity, overreliance on statistical models, extrapolations in the absence of primary data, and difficulty in attributing mortality in cases with multiple causes [49,50,51]. Five, the progress toward SDG 3.4 was measured using the percentage change in diabetes-related deaths, which does not account for changes in population size or age structure.

## 5. Conclusions

The projected rise in diabetes mortality presents a critical challenge for public health systems worldwide. Achieving progress toward SDG 3.4, which aims to reduce mortality from diabetes, will require a multifaceted approach, including enhanced preventive strategies, early diagnosis, and improved disease management. Forecasting models, such as those employed in this study, are crucial in guiding policy decisions by providing data-driven insights into future mortality trends. By integrating predictive analytics into public health planning, policymakers and healthcare providers can implement targeted interventions to curb diabetes-related deaths, reduce healthcare burdens, and improve long-term health outcomes.

## Figures and Tables

**Table 1:** Forecasted median number of deaths (in thousands except type 2) from diabetes in 2030 and associated 95% prediction interval by type of diabetes, age groups, WHO regions, and World Bank income classification.

Category	ARIMA	GAM	GLM	Prophet	NSE (UW)	NSE (W)
Type of diabetes						
Type 1	88.3 (81.9, 94.7)	90.3 (86.0, 94.6)	82.9 (81.4, 84.5)	92.9 (85.6, 100.3)	86.5 (83.6, 89.2)	80.1 (76.9, 83.3)
Type 2 *	1.9 (1.8, 2.1)	2.0 (1.9, 2.1)	1.7 (1.6, 1.7)	1.9 (1.8, 2.0)	1.5 (1.3, 1.6)	1.5 (1.3, 1.6)
Age Group						
<5	2.23 (1.82, 2.64)	1.94 (1.53, 2.34)	2.28 (81.33, 84.46)	92.87 (85.6, 100.27)	2.36 (2.2, 2.52)	2.36 (2.2, 2.52)
5–14	2.42 (1.61, 3.23)	1.96 (1.48, 2.44)	2.55 (2.42, 2.68)	2.6 (2.11, 3.09)	2.46 (2.18, 2.8)	2.46 (2.18, 2.8)
15–49	137.5 (121.7, 153.2)	134.5 (122.8, 146.1)	140.4 (137.3, 143.6)	139.6 (130.0, 149.1)	128.1 (124.7, 131.1)	128 (124.7, 131.0)
50–69	761.6 (614.4, 908.8)	773.1 (714.5, 831.7)	657 (637.1, 676.9)	777.9 (750.5, 801.5)	646.8 (594, 705.8)	647.7 (593, 706.4)
70+	1178.7 (995.7, 1361.8)	1188.4 (1127.9, 1249)	996.9 (981.2, 1012.6)	1120.7 (1067.5, 1177.3)	887.4 (821, 967.8)	886.5 (820.1, 963.8)
WHO Region						
AFR	209.2 (614.4, 908.8)	205.8 (714.5, 831.7)	205.6 (637.1, 676.9)	212.3 (750.5, 801.5)	186.9 (594, 705.8)	187 (593, 706.4)
AMR	383.2 (347.7, 418.7)	423.9 (395.9, 451.9)	343.2 (336.4, 350)	398.2 (377, 422.9)	307.8 (286.9, 329.2)	308 (287.2, 329.3)
EMR	174 (147, 201)	175.2 (164.8, 185.5)	149.9 (145.1, 154.7)	174.6 (167.9, 180.9)	151.5 (136.9, 165.3)	151.4 (136.9, 165.4)
EUR	206.3 (191.7, 221)	208.8 (187.2, 230.4)	195 (190.7, 199.4)	224.3 (212.5, 236.7)	196.6 (185.7, 207.1)	196.4 (185.7, 207.1)
SEAR	646 (451.4, 840.7)	654.4 (586.7, 722.1)	568 (551.1, 584.9)	654.4 (630.2, 677.8)	530.6 (474.4, 582.4)	530.2 (474.2, 582.3)
WPR	357.2 (316.7, 397.7)	394.7 (364.3, 425.2)	329.5 (322.7, 336.3)	378.7 (328.5, 436.6)	292.8 (267.4, 317.4)	292.6 (267, 317.1)
World Bank Income Classification						
High-Income	299.7 (614.4, 908.8)	336.9 (714.5, 831.7)	265.7 (637.1, 676.9)	300.2 (750.5, 801.5)	245.7 (594, 705.8)	245.9 (593, 706.4)
Low-Income	127.5 (116.7, 138.2)	128.7 (125.4, 132)	107.9 (105.3, 110.4)	123.5 (119.5, 127.6)	99.2 (89.6, 109.7)	99.2 (89.7, 109.7)
Low-Middle	930.1 (721.3, 1139)	938.8 (860, 1017.5)	828.8 (806.2, 851.3)	941.6 (909.6, 973.8)	765.9 (690.9, 838.3)	766.6 (691.3, 838.2)
Upper-Middle	684.2 (528.8, 839.5)	768.2 (673.7, 862.7)	595 (583, 607)	682.6 (645.8, 722.2)	532.7 (480.2, 585.1)	533.7 (481.4, 584)

* in million; NSE (UW): n-sub-epidemic ensemble unweighted; NSE (W): n-sub-epidemic ensemble weighted; a 95% prediction interval provides a range that is expected to contain the value of a single future observation, with 95% probability, given the model and its assumptions; AFR: African region, AMR: region of the Americas, EMR: Eastern Mediterranean region, EUR: European region, SEAR: Southeast Asia region, WPR: Western Pacific region.

## Data Availability

The original data presented in the study are publicly available diabetes mortality data from Our World in Data (OWID) through https://ourworldindata.org/data (accessed on 10 April 2025). The original data source is the Institute for Health Metrics and Evaluation, Global Burden of Disease (GBD) 2019.

## Supplementary Materials

The following supporting information can be downloaded at: https://www.mdpi.com/article/10.3390/jcm14103364/s1. References [52,53,54,55,56,57,58,59,60,61,62,63,64,65,66,67,68,69,70,71,72,73] are cited in Supplementary Materials.
