# Supplementary material for: The Future Diabetes Mortality: Challenges in Meeting the 2030 Sustainable Development Goal of Reducing Premature Mortality from Diabetes
Source: J Clin Med. 2025 May 12;14(10):3364. doi: 10.3390/jcm14103364 (PMC12112454; doi:10.3390/jcm14103364)
Supplement: Supplementary file 1 [file jcm-14-03364-s001.zip › jcm-3611533-supplementary.pdf]

Supplementary Materials

S1. Auto-Regressive Integrated Moving Average Models (ARIMA)

The ARIMA model is commonly employed in forecasting financial [52] and weather trends [53,54] and has become a standard benchmark in disease forecasting [25,26,55–57]. ARIMA model performs well for short-term predictions when mortality follows consistent patterns but is less effective at capturing sudden shifts in mortality trends due to healthcare interventions.

ARIMA model consists of three parts: the auto-regression (AR) part involving regressing on the most recent values of the series, the moving average (MA) of error terms occurring contemporaneously and at previous times, and the integration (I) or differencing to account for the overall trend in the data and to make the time series stable. Mathematically, an ARIMA (p, d, q) process is given by:

$$\phi(B)(1-B)^d y_t = c + \theta(B)\epsilon_t, \qquad \text{(S1)}$$

where y_t denotes the number of diabetes deaths at time t, B denotes the backshift operator implying $By_t = y_{t-1}$ and $B(By_t) = B^2 y_t = y_{t-2}$, etc., $\phi(\mathrm{B}) = 1 - \phi_1 B - \cdots - \phi_p B^p$ and p is the order of the AR model, d is the degree of differencing, $\theta(B) = 1 + \theta_1 B + \cdots + \theta_q B^q$ and the q is the order of the MA model [58]. With this notation, $\phi(\mathrm{B})y_t = y_t - \phi_1 y_{t-1} - \cdots - \phi_p y_{t-p}$, $\theta(B)\epsilon_t = \epsilon_t + \theta_1 \epsilon_{t-1} + \cdots + \theta_q \epsilon_{t-q}$, and $(1-B)^d$ means conducting the differencing d times. The *auto.arima* function in the R package "forecast" was used to select values for p, d and q and build the model [59] and the forecast function in the R package "forecast" was used for forecasting [60]. Any negative predicted values were truncated at zero.

S2. Generalized Additive Models (GAM)

Generalized additive models are an extension of generalized linear models that include a sum of unknown smooth functions of some covariates [20]. They can capture non-linear trends while maintaining similar model explainability and simplicity levels as seen with generalized linear models [61]. GAMs contribution relative to other methods is in the following ways: In contrast with GLMs, GAMs are more flexible in dealing with non-linear trends. Compared to ARIMA, GAMs capture long-term patterns better. Also, GAMs are more interpretable than the decomposition approach offered by Prophet [61]. Specific to our study with time as the only covariate, assuming that y_t has a normal distribution, our GAM is given as [61]:

$$y_t = \beta_0 + s(t) + \epsilon_t, \qquad \text{(S2)}$$

where $s(.)$ remains an unknown smooth function of time, and $\epsilon_t \sim N(0, \sigma^2)$. We fitted this model (2) using the gam function available in the R package "mgcv". In this package, smooth function s(.) is represented by means of basis functions, which are elements of a linear combination forming more complex functions. Default settings in the "mgcv" package employ basis splines, and piecewise polynomial functions [28]. Specifically,

$$s(t) = \sum_{k=1}^{k} \beta_k b_k(t), \qquad \text{(S3)}$$

where$\{b_k(.)\}$ represent the basis functions, $\{\beta_k\}$ are the expansion coefficients to be estimated, and k is the number of basis functions [61]. The value of parameter k was flexible based on the amount of calibration data for each forecasting period. A discrete penalty was imposed on the basis coefficients to control the degree of smoothness, and the model was adjusted using penalized least squares. It was the generalized cross-validation criterion that optimally set the smoothness tuning parameter [28]. A more detailed description of the model fitting methodology can be found in [56,62], and the associated *predict* function was used for forecasting [63]. The values were truncated at zero, if predicted values were negative.

S3. Facebook's Prophet Model

Initially designed for business-related forecasting, Meta-Facebook's Prophet model [23] has recently been applied more frequently across multiple fields, including non-communicable diseases

research. Prophet meant for time series data featuring prominent trends, seasonality, and possible change points. It automatically detects change points, which are significant shifts in trends, and accommodates missing data and outliers. It is best suited for long-term forecasts where trends may change over time. Specifically, the model has produced Diabetes prevalence forecasts [64]. The model's primary assumption is that it is "decomposable". Therefore, the trend $y(t)$ is decomposed into three pieces plus an error term. Thus, the model has the following form:

$$y(t) = g(t) + s(t) + h(t) + \epsilon_t \quad \text{(S4)}$$

where $g(t)$ is a non-periodic component used for modeling the overall trend, $s(t)$ is a periodic component used for modeling periodic changes over time, and $h(t)$ is an irregular events component used for modeling irregular changes (e.g. holidays or similar events). The component ϵ_t is an error of the model at time t. We used the default setting of the R *prophet* function from the "prophet" package where the model has been implemented as given in [65]. The *predict* function was used for forecasting from the model fit [23]. The values were truncated at zero, if predicted values were negative. A more detailed description of the model fitting methodology can be found in [56,65].

S4. The n-sub-epidemic modeling framework

The n-sub-epidemic framework employs multiple epidemic trajectories modeled as the aggregation of overlapping and asynchronous sub-epidemics [25,66]. Unlike simpler models, this approach captures important features such as the plateau stage, followed by rise in mortality for certain population groups or geographies. We merged forecasts from several higher performing models to create more trustworthy predictions accompanied with achievable uncertainty outlines. For this analysis, a single sub-epidemic follows a 3-parameter generalized-logistic growth model (GLM), which has displayed competitive performance in the context of varying infectious diseases, including Zika, Ebola, and COVID-19 [67–70]. Further details regarding the structure of the 3-parameter GLM model can be found in the supplementary file (Additional methods A.1).

An n-sub-epidemic trajectory is comprised of n overlapping sub-epidemics and is given by the following system of coupled differential equations:

$$\frac{dC_i(t)}{dt} = C_i{}'(t) = A_i(t) r_i C_i{}^{p_i}(t)\left(1 - \frac{C_i(t)}{K_{0_i}}\right). \quad \text{(S5)}$$

The incidence curve of diabetes deaths is given by $\frac{dC_i(t)}{dt}$, where $C_i(t)$ tracks the cumulative number of diabetes-related deaths for sub-epidemic i. The parameters that characterize the shape of the i-th sub-epidemic are given by (r_i, p_i, K_{0_i}), for $i = 1, \ldots, n$. The parameter r is the growth rate per unit of time, must be positive, and parameter K_0 represents the final outbreak size. The "scaling of growth" parameter $p \in [0,1]$ allows the model to capture early sub-exponential and exponential growth patterns. If $p = 0$, this equation describes a constant number of new cases over time, while $p = 1$ indicates that the early growth phase is exponential. Intermediate values of p $(0 < p < 1)$ describe early sub-exponential (e.g., polynomial) growth dynamics.

Th value n represents the number of sub-epidemics considered in the epidemic's trajectory. When $n = 1$, the sub-epidemic model is equivalent to the 3-parameter GLM model. However, when $n > 1$, we employ the indicator variable$A_i(t)$, to model the onset timing of the$(i+1)_{th}$ sub-epidemic, where $(i+1) \leq n$. Therefore, the $(i+1)_{th}$ sub-epidemic is triggered when the cumulative curve of the i_{th} sub-epidemic exceeds the case threshold value, C_{thr} (i.e., $C_{thr} \leq K_{0_i}$). Thus, we have

$$A_i(t) = \begin{cases} 1, C_{i-1}(t) > C_{thr} \\ 0, Otherwise \end{cases} \; for\; i = 2, \ldots, n\,, \quad \text{(S6)}$$

where $A_1(t)$ =1 for the first sub-epidemic. Therefore, $3n + 1$ parameters are needed to model an n-sub-epidemic trajectory for $n > 1$. This analysis considers a maximum of two sub-epidemics in the n-sub-epidemic trajectory $(n \leq 2)$. The initial number of diabetes deaths is given by $C_1(0) = I_0$, where I_0 is the initial number of cases observed in the data. The cumulative curve of the n-sub-epidemic trajectory is given by:

$$C_{tot}(t) = \sum_{i=1}^{n} C_i(t). \quad \text{(S7)}$$

Overall, the modeling framework can be applied to diverse epidemic patterns including those characterized by multiple peaks and extended high-deaths level plateaus.

S5. Parameter estimation and model selection

We employed the nonlinear least squares method given in [66] to estimate the model parameters by fitting the model solution to the observed Diabetes deaths data. Subsequently, we selected the top-ranked sub-epidemic by the corrected Akaike Information Criterion (AIC_c) values of the set of best-fit models based on two sub-epidemics. The AIC_c is given by [71,72]:

$$AIC_c = n_d log(SSE) + 2m + \frac{2m(m+1)}{n_d-m-1} \quad \text{(S8)}$$

where $SSE = \sum_{j=1}^{n_d}(f(t_j,\hat{\Theta}) - y_{t_j})^2$, m is the number of model parameters, and n_d is the number of data points. Parameters from the above formula for AIC_c are estimated from the nonlinear least-squares fit, which implicitly assumes normal distribution for error. Additional information regarding the parameter estimation process can be found in the supplementary file (Additional methods A.2).

S6. Parametric Bootstrapping

The n-sub-epidemic framework quantifies parameter uncertainty for the best fit-model, $f(t,\hat{\Theta})$, through a bootstrapping approach described in [73], which allows the computation of standard errors and related statistics without closed-form solutions. Additionally, we ran the calibrated model forward in time to generate forecasts (i.e., 11 years ahead) with quantified uncertainty, employing the same parametric bootstrapping method presented in [73] and discussed further in supplementary file (Additional methods A.3). We used 300 bootstrap realizations for this analysis to characterize parameter and forecast uncertainty.

S7. Constructing ensemble n-sub-epidemic models

We generated ensemble models from both the unweighted (equally weighted top-ranking models) and weighted combination of the two highest-ranking sub-epidemic models (i.e., top- and second-ranked) as deemed by the AIC_{c_i} for the *i-th* ranked model where $AIC_{c_1} \leq \cdots \leq AIC_{ci}$ and i = 1, 2 . Specifical details regarding weighted and unweighted calculations can be found in supplementary file (Additional methods A.4). The prediction intervals based on the ensemble models were obtained using the bootstrapping approach from above.

Additional Methods

1. Three-Parameter Generalized Logistic Growth Model (GLM)

The three-parameter GLM model is given by the following relationship:

$$\frac{dC(t)}{dt} = C'(t) = rC^p(t)\left(1 - \frac{C(t)}{K_0}\right), \quad (1)$$

where $C(t)$ denotes the cumulative diabetes deaths at time t and $\frac{dC(t)}{dt}$ describes the incidence curve of diabetes deaths over time t. The parameter r, the growth rate per unit of time, remains positive, and parameter K_0 represents the final outbreak size. The "scaling of growth" parameter $p \in [0, 1]$ allows the model to capture early sub-exponential and exponential growth patterns. If $p = 0$, this equation describes a constant number of new cases over time, while $p = 1$ indicates that the early growth phase is exponential. Intermediate values of p ($0 < p < 1$) describe early sub-exponential (e.g., polynomial) growth dynamics.

2. Parameter Estimation for Ensemble Sub-Epidemic Models

Let the time series of new incident diabetes deaths used in model calibration be denoted as $y_{t_1}, y_{t_2}, \dots, y_{t_{n_d}}$ where t_j, $j = 1,2,\dots,n_d$, are the time points for the time series data, and n_d is the

number of observations. Using these case series, we estimated a total of $3n + 1$ model parameters, namely $\Theta = (C_{thr}, r_1, p_1, K_{0_1}, \ldots, r_n, p_n, K_{0_n})$ when n, the number of sub-epidemics, is greater than 1. Let $f(t, \Theta)$ denote the expected curve of new diabetes deaths of the epidemic's trajectory. Using the nonlinear least squares method, we considered different values of C_{thr} by discretizing its range of plausible values. For each C_{thr}, we searched for the set of parameters $\hat{\Theta}$ that minimized the sum of squared differences between the observed data $y_{t_1}, y_{t_2} \ldots y_{t_{n_d}}$ and the model mean $f(t, \Theta)$. We then selected C_{thr} and other associated parameters based on results that led to the smallest sum of squared errors. That is, $\Theta = (C_{thr}, r_1, p_1, K_{0_1}, \ldots, r_n, p_n, K_{0_n})$ was estimated by $\hat{\Theta} = arg\ min\ \sum_{j=1}^{n_d}(f(t_j, \Theta) - y_{t_j})^2$.

3. Parametric Bootstrapping

We quantified parameter uncertainty using the best-fit model, $f(t, \hat{\Theta})$, to generate B-times replicated simulated datasets of size n_d, where the observation at time t_j is independently sampled from a normal distribution with mean $f(t_j, \hat{\Theta})$ and variance $\frac{\sum_{j=1}^{n_d}(f(t_j,\hat{\Theta})-y_{t_j})^2}{n_d - m}$, where m is the number of parameters with $m = 3$ for 1 sub-epidemic (i.e., $n = 1$) and $m = (3n) + 1$ for $n > 1$. Then, we refit the model to each B simulated dataset to re-estimate each parameter. The new parameter estimates for each realization are denoted by $\hat{\Theta}_b$ where $b = 1,2, \ldots, B$. Using the sets of re-estimated parameters $(\hat{\Theta}_b)$, the empirical distribution of each estimate can be characterized, and the resulting uncertainty around the model fit was obtained from $(t, \hat{\Theta}_1), f(t, \hat{\Theta}_2), \ldots, f(t, \hat{\Theta}_B)$.

4. Constructing Ensemble n-Sub-Epidemic Models

We generated ensemble models from both the unweighted (equally weighted top-ranking models) and weighted combination of the two highest-ranking sub-epidemic models (i.e., top- and second-ranked) as deemed by the AIC_{c_i} for the i-th ranked model, where $AIC_{c_1} \leq \cdots \leq AIC_{c_I}$ and $i = 1, \ldots, I$. We compute the weight w_i for the i-th model, $i = 1, \ldots, I$, as follows:

$$w_i = \frac{l_i}{l_1 + l_2 + \cdots + l_I}, \quad (2)$$

where l_i is the relative likelihood of model i, which is given by $l_i = e^{((AIC_{min} - AIC_i)/2)}$ [1], and hence $w_I \leq \cdots \leq w_1$. For the unweighted model, we use the same weight $w_i = 1/I$ for all models, $i = 1, \ldots, I$.
